# Epilepsy in Patients with Cerebral Radiation Necrosis: A Scoping Review

**DOI:** 10.3390/medicina62030561

**Published:** 2026-03-18

**Authors:** Paul V. Q. Laman, Josien C. C. Scheepens, Lente L. Kroon, Maaike J. Vos, Dieta Brandsma, Johan A. F. Koekkoek

**Affiliations:** 1 Department of Neurology, Leiden University Medical Center, 2333 ZG Leiden, The Netherlands; p.v.q.laman@lumc.nl (P.V.Q.L.); j.c.c.scheepens@lumc.nl (J.C.C.S.); m.j.vos@lumc.nl (M.J.V.); 2 Department of Neuro-Oncology, Netherlands Cancer Institute-Antoni van Leeuwenhoek, 1066 CX Amsterdam, The Netherlandsd.brandsma@nki.nl (D.B.); 3 Department of Neurology, Haaglanden Medical Center, 2501 CK The Hague, The Netherlands

**Keywords:** cerebral radiation necrosis, brain necrosis, treatment-induced brain necrosis, radiation-induced brain toxicity, epilepsy, seizures, radiotherapy

## Abstract

*Background and Objectives*: Cerebral radiation necrosis (CRN) is a delayed complication of radiation therapy (RT), that can appear either as radiological findings without clinical symptoms (i.e., asymptomatic CRN) or symptomatic CRN (sCRN). There is currently a knowledge gap regarding CRN-induced epilepsy, a potentially severe manifestation of sCRN. We aim to give a comprehensive overview of the existing literature on CRN-induced epilepsy, including its prevalence, potential risk factors, and treatment options. *Materials and Methods*: A scoping analysis was performed according to the PRISMA scoping review guidelines. We searched within PubMed and Embase databases and identified relevant clinical studies for inclusion related to CRN-induced epilepsy, based on predefined criteria. *Results*: In total, 24 studies were identified. CRN-induced epilepsy was a primary outcome in three studies. In the 21 remaining studies, epilepsy was an exploratory outcome or described as part of a small case series. The studies covered various topics in relation to CRN-induced epilepsy, such as the overall clinical manifestations of CRN, the optimization of RT practices to minimize toxicity and improve outcomes, and the effectiveness of laser interstitial thermal therapy (LITT) and bevacizumab. Considerable heterogeneity was seen across studies, particularly concerning the primary tumor types, used definition of CRN and applied RT practices. *Conclusions*: Epilepsy is a serious clinical symptom in patients with sCRN. However, the current literature is too limited to draw meaningful conclusions regarding its prevalence, risk factors and management. Future research in patients with sCRN should prioritize the evaluation of clinical response to different treatment strategies with particular attention to seizure control.

## 1. Introduction

Radiotherapy (RT) is a common, non-invasive approach to treat primary and secondary brain tumors, next to other antitumor treatments, such as surgery, chemotherapy and targeted treatment [1,2]. RT, typically delivered as external beam radiation therapy (EBRT), includes modalities such as stereotactic radiotherapy (SRT) and whole-brain radiotherapy (WBRT). Local RT treatment improves intracranial disease control and overall survival in patients with brain tumors [3]. Although RT is proven effective and generally considered to be safe, serious complications can occur as a result of radiation-induced damage, including cerebral radiation necrosis (CRN) [1,2,3,4,5,6,7].

CRN is formally defined by the Common Terminology Criteria for Adverse Events (CTCAE) version 5.0 as “a necrotic process occurring in the brain and/or spinal cord” and falls within the category of delayed-onset radiation damage, generally occurring from three months to several years after RT [2,4]. The incidence rate of both asymptomatic and symptomatic CRN (sCRN) together is estimated to range from 5 to 25%. Here, higher RT doses, prior RT treatment, larger target volume, and concurrent chemotherapy or immunotherapy were associated with an increased risk of CRN development [1,2,3,4,5,6,7]. Moreover, specific tumor histology and certain areas of the brain can be more susceptible to the development of CRN [1,2,3,4,5,6,7]. For example, it has been suggested that brain metastases (BMs) originating from lung cancer, HER2-amplified breast cancer, and BRAF V600 wild-type melanoma have been associated with an increased risk of CRN after RT [6].

The diagnosis of CRN remains challenging. Various post-irradiation syndromes are recognized clinically, but definitions are frequently inconsistent. Pseudoprogression for instance, a transient effect usually occurring before CRN (i.e., less than three months after RT), is often incorrectly used interchangeably, misclassifying it as CRN [8,9]. Furthermore, CRN can be difficult to distinguish from tumor recurrence in patients with a history of malignant brain tumors [1,2]. On conventional Magnetic Resonance Imaging (MRI), the features of CRN and tumor recurrence often overlap, as both can present with contrast enhancement. Additionally, increased perilesional edema, while commonly associated with the tumor itself, particularly in BMs and gliomas, can also be a manifestation of CRN. This makes conventional MRI insufficiently reliable to diagnose CRN [1]. Imaging modalities such as MR perfusion, MR spectroscopy and fluoroethyltyrosine positron emission tomography (FET-PET) may help to facilitate the diagnosis of CRN. The gold standard remains a histopathological confirmation. However, this diagnostic modality does not always provide a precise diagnosis, as tissue necrosis and viable tumor cells may both be present in the sample, and robust pathological criteria for CRN are lacking. Moreover, tissue samples are not always easily obtained, particularly from deep-seated lesions or those located in functional areas, resulting in sampling error and risk of complications for the patient [1].

CRN may be detected solely on imaging without associated symptoms, or it may manifest with a range of clinical symptoms, including epilepsy. Beyond impairing cognitive and daily functioning, which can significantly affect patients’ quality of life, seizures may cause life threatening complications, such as a status epilepticus, serious injuries, or sudden unexpected death [10,11,12,13,14]. Evidence on the prevalence and optimal management of CRN-induced epilepsy is scarce, with uncertainties regarding the effectiveness of available treatments, such as corticosteroids, antiseizure medication (ASM) and bevacizumab [10,11,12]. In this scoping review, we aim to critically synthesize the literature on sCRN and its association with epilepsy. This involves evaluation of the methodological approaches of the included studies employed to define, measure, and report CRN-induced epilepsy, and the identification of knowledge gaps to guide future research and treatment.

## 2. Methods and Design

### 2.1. Literature Search

Our study adhered to the PRISMA guidelines for scoping reviews (Supplementary S1) [15,16]. With the expertise of an experienced librarian, we designed two literature search queries for the bibliographic databases PubMed and Embase, covering relevant terms for CRN and epilepsy (Supplementary S2). We conducted our search on 16 May 2025, and subsequently exported the results to Zotero (version 7.0.16) where duplicates were removed.

### 2.2. Literature Selection

We included peer-reviewed studies in our review based on pre-defined criteria. We adhered to the following inclusion criteria for both abstract and full text screening: Studies needed to (1) specifically address seizures because of sCRN, (2) involve human participants, (3) include a sample size ≥2, and (4) be published in the English language. We excluded studies if: (1) they involved a non-original article (e.g., conference abstracts, study protocols, or reviews), (2) a full-text version was not available, and (3) they only included pediatric patients. Although reviews were excluded from the final selection, their reference lists were manually screened to identify potentially relevant studies.

One of the co-authors (P.V.Q.L.) screened titles and abstracts, excluding studies that did not meet the inclusion criteria. In the second stage, the same co-author screened the full texts using the same criteria. Afterwards, a second reviewer (J.C.C.S.) verified all excluded titles and abstracts and full-texts, and inter-rater reliability was assed using Cohen’s Kappa. Any discrepancies were resolved through discussion among the co-authors (P.V.Q.L., J.A.F.K., and J.C.C.S.). Finally, expert consultation (J.A.F.K and D.B) was sought to identify potentially missing studies, ensuring coverage of all relevant studies.

### 2.3. Data Extraction and Synthesis

We determined the data items to be extracted through an iterative process involving collaboration and input from P.V.Q.L, J.A.F.K., and J.C.C.S. Next, P.V.Q.L. charted the data from the full-text articles using a Microsoft Excel form. Here, each row corresponds to a single study with the columns denoting the extracted information (Supplementary S3). We first extracted general article characteristics from the selected studies including authors, year of publication, country of origin, study design, study approach, sample size, sex distribution, population age, and primary diagnosis. Secondly, we assessed the scope and methodological approach used in each study to define, measure, and report CRN-induced epilepsy. This assessment included the extraction of the study aim, article focus, number of CRN patients, number of patients with CRN-induced epilepsy, CRN diagnostic modality, time interval between RT and CRN diagnosis, RT modality, seizure type and seizure focus (Table 1). To define article-focus, studies were categorized as primary, secondary, or exploratory. Primary studies focused on CRN-induced epilepsy as the main outcome. Secondary studies listed it as an outcome in the method section, but not as the main focus. Exploratory studies mentioned it only in the results, representing descriptive or post hoc analyses. Finally, small case series (cohort size 2–10 patients) were classified separately. Although these studies may provide valuable descriptive insights, their limited sample size justified distinct categorization.

## 3. Results

### 3.1. Selected Literature

**Figure 1:**
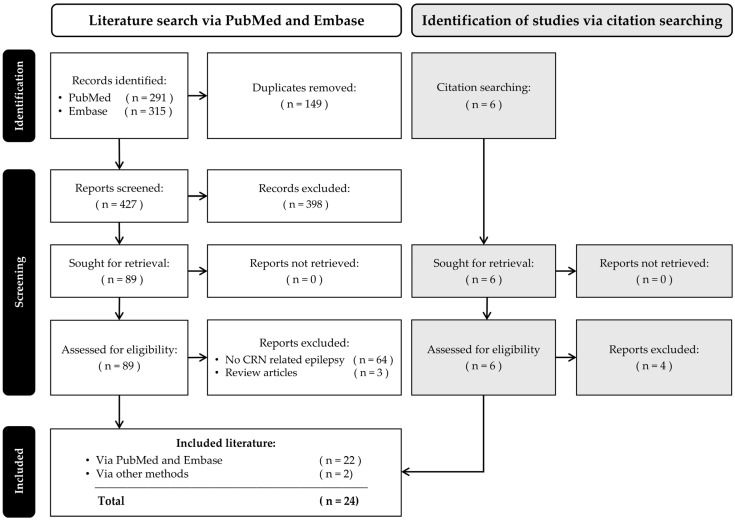
PRISMA flow diagram depicting the consecutive steps of the scoping review.

For the phases of the search and selection process, we refer to the PRISMA flow diagram (Figure 1) [15,16]. Our search identified 606 studies in PubMed and Embase. After removal of 149 duplicates in Zotero, 457 unique abstracts were screened, leading to 89 articles for further full-text assessment (Cohen’s Kappa = 0.82). Finally, the full-text screening resulted in 22 original articles being included. During the screening process, we identified an additional six studies through citation searching, of which two were included in the final selection (Cohen’s Kappa = 0.91).

### 3.2. General Article Characteristics

The 24 selected articles were published from 1987 to 2025. Ten were conducted in the USA (41.7%), five in China (20.8%), three in Italy (12.5%), and the remaining 6 in other countries (25.0%). All studies had an observational design with a longitudinal approach. The median cohort size was 13 (range 2–532) and the mean percentage of female participants was 47% (range 0–100%). With regard to primary diagnosis, seven studies included patients with BMs, four studies included patients with nasopharyngeal carcinomas (NPCs), and three studies included patients with meningiomas. Additionally, five individual studies focused on pituitary adenoma, mesial temporal lobe epilepsy (MTLE), astrocytoma, medulloblastoma and lymphoma respectively. The remaining five studies included a mix of intracranial and head and neck pathologies (Table 2).

### 3.3. Scope of Literature and Study Heterogeneity

The included studies focused on a wide range of topics, including the treatment of sCRN (with bevacizumab, laser interstitial thermal therapy (LITT), or surgery), the optimization of RT practices to minimize toxicity and improve clinical outcomes, and a general exploration of the clinical spectrum of CRN. The majority of included small case series, and the larger studies by Black et al. (2013) [20] and Di Stefano et al. (2019) [24], described stroke-like events associated with delayed effects of brain irradiation. These included stroke-like migraine attacks after radiation therapy (SMART) syndrome, peri-ictal pseudoprogression (PIPG), and acute late-onset encephalopathy after radiation therapy (ALERT) syndrome. We included these studies, as these syndromes do present seizure-like phenomena and may be regarded as a late form of sCRN (Table 3).

Besides the variety in study aims and general study characteristics, we found a large heterogeneity in the used definition of CRN and RT modalities across the included studies. In most studies, the diagnosis of CRN was based on clinical and/or radiological characteristics (*n* = 18), while a minority of studies (*n* = 5) relied on histopathological confirmation. The study of Sujijantarat et al. (2020) [37], which compared LITT and bevacizumab for the treatment of sCRN, confirmed CRN histopathologically in the LITT treated subgroups, whereas the bevacizumab treated subgroup was diagnosed clinically and radiologically. Five studies based their diagnosis of sCRN on prespecified criteria within their methodology, such as defined radiological features of CRN or a minimal time interval between RT and CRN for inclusion [10,24,25,36,37]. Across all studies, sCRN was diagnosed between 3- and 240 months following RT, with a median interval of 24 months. This long median interval can be explained by the inclusion of studies on SMART syndrome, which could be described as a late-onset form of CRN. Interestingly, the article of Hou et al. (2024), who evaluated epilepsy as a primary outcome, did not specify how sCRN was diagnosed [12]. Overall, the median number of CRN-induced epilepsy cases per study was two (range 1–65), with ten studies only reporting a single case (Table 3). Twelve studies specified the seizure type, and thirteen reported the brain region affected by radiation toxicity. Notably, Huang et al. (2020) [10] elaborated on the association between CRN location in the brain and epilepsy, yet they limited their analysis to sCRN in the temporal lobe (Table 4).

A wide range of RT strategies and modalities were used in the included studies. Overall, only three studies failed to specify the applied RT treatment strategy. For clarity, we grouped certain strategies under the category of conventional RT. This category includes modalities explicitly described as such, as well as those referred to as traditional RT, two-dimensional RT, or simple RT without any further specification. The study by Calafiore et al. (2025) reported the use of fractionated EBRT, which included treatments such as three-dimensional conformal RT (CRT), intensity-modulated radiotherapy (IMRT), or volumetric modulated arc therapy (VMAT) [22]. Similarly, Shah et al. (2025) [32] compared conformal partial brain (CPB) irradiation, a form of three-dimensional CRT, with SRT. In CRT, radiation beams are shaped to match the dimensions of the tumor, whereas in SRT this is not the case. In eight studies, the applied RT modality could be identified for individual patients with CRN-induced epilepsy [17,22,28,30,34]. As these studies included one or two patients with CRN-induced epilepsy, we report the treatment for each individual (Table 4).

### 3.4. Data on CRN-Induced Epilepsy

Only three included studies focused on CRN-induced epilepsy as a primary outcome, aiming to directly study the prevention and management of sCRN and epilepsy [10,11,12]. Hou et al. (2024) [12] studied the use of bevacizumab for the treatment of epilepsy following SRT, and reported sustained seizure freedom, improved quality of life, and reduced seizure severity and need for ASM. Of the 41 patients treated with 7.5 mg/kg bevacizumab every 21 days for eight cycles, five patients (12.2%) achieved sustained seizure freedom through a 12-month follow-up period. Furthermore, a reduction in baseline seizure frequency was seen in an additional 12 patients (29.3%). Huang et al. (2020) [10] developed a nomogram to predict epilepsy in patients with CRN, which could help clinicians to identify patients at risk. Among 302 NPC patients with CRN, they identified several significant predictors for CRN-induced epilepsy. Higher maximum radiation dose to the temporal lobe, larger RN volume, increased serum creatine phosphokinase (CK), lower cholesterol, a history of hypertension and/or diabetes, and male sex were all linked to an increased risk of epilepsy. Maximum radiation dose to the temporal lobe, larger RN volume, and increased serum CK were the most significant predictors, each with a *p*-value < 0.001. Finally, Rong et al. (2017) [11] investigated statin use for preventing CRN-induced epilepsy in 532 NPC patients and found that statin use was associated with a lower risk of epilepsy (6.8% vs. 18.5%; *p* = 0.007). Interestingly, the proportion of patients developing CRN was similar in the statin and non-statin group (92.0% vs. 87.8%, respectively). This suggests that statins may not prevent the development of CRN, but rather influence downstream inflammatory or epileptogenic processes following CRN. Altogether, the mechanism by which statins may reduce epilepsy incidence in patients with sCRN requires further research.

None of the included studies investigated CRN-induced epilepsy as a secondary outcome. Hence, the remaining 21 studies included CRN-induced epilepsy as an exploratory outcome or described it as part of a small case series. Most of the case series offered descriptive accounts of patients suffering from SMART syndrome. Even in the study conducted by Woo et al. (1987) [35], which describes seven individuals with proclaimed CRN, symptoms occurred at a median of 60 months after RT, possibly more closely resembling SMART. The relatively large number of case-series published on this topic reflects the rarity of SMART and the difficulty of studying it on a larger scale. Next, while the majority of included exploratory studies focused on the optimization of RT practices, evidence on the association between RT modalities and epilepsy development remains limited. For instance, the study of Shah et al. (2025) [32] indicates that CPB irradiation decreases the risk of CRN compared to SRT, thereby potentially reducing the risk of epilepsy. However, due to the small sample size (*n* = 55), these findings remain preliminary. Similarly, in studies covering the treatment of sCRN, evidence on the relation between sCRN treatment and risk of epilepsy is limited. For instance, Sujijantarat et al. (2020) [37] compared LITT with bevacizumab for treating CRN in patients with BM and found that LITT was associated with longer overall survival and greater long-term lesion volume reduction than bevacizumab. However, their study did not determine conclusively whether LITT is more effective than bevacizumab in controlling CRN-related epilepsy. One study, by Chan et al. (2023) [23] did report that seizure prevalence decreased from 34.4% to 12.0% in the first month following LITT in a population of 90 histopathologically confirmed CRN patients. Three months following LITT, seizure prevalence decreased to 7.9%. In reaction to remission of seizures, ASM dosages could be tapered down or stopped in 64% of the patients with epilepsy. As these promising results are only shown in a single study, effects of LITT on seizure outcomes warrant further investigation.

Finally, findings on incidences of CRN-induced epilepsy across studies must be interpreted with caution, due to small sample sizes and heterogeneous patient populations. Among the studies with >10 CRN patients that did not restrict inclusion to specific subgroups (e.g., patients with epilepsy), the reported proportion of patients with sCRN developing epilepsy ranges from 8% to 36%.

## 4. Discussion

Although a rare manifestation of sCRN, epilepsy represents a serious clinical symptom that can profoundly impair a patient’s quality of life. In the context of an increasing survival among subgroups of patients with brain tumors, studying CRN and its associated symptoms is of growing significance as a larger proportion of patients may experience the delayed effects of RT.

Overall, this scoping review underscores the limited body of evidence, indicating that our current understanding of the prevalence, risk factors, and management of CRN-induced epilepsy remains scarce. We identified 24 studies, published between 1987 and 2025, that reported on CRN-induced epilepsy. Notably, 10 of these studies reported only a single patient with CRN, illustrating how limited available literature is. In total, the majority of the included studies discussed CRN-induced epilepsy in an exploratory context. Only three studies focused on CRN-induced epilepsy as a primary endpoint, two of which were conducted by the same research group using non-overlapping populations [10,11]. Across the 24 identified studies, substantial variability was observed in reporting practices and methodological approaches. Therefore, it should be acknowledged that most findings regarding CRN and epilepsy occurrence, as shown in Table 3, may not accurately reflect the true clinical incidence of these conditions. Most studies described their method of CRN diagnosis, predominantly relying on clinical and/or radiological findings, and less frequently on confirmation by histopathology. However, diagnostic criteria for CRN, such as imaging characteristics, associated symptoms, and the minimal time interval between RT and CRN diagnosis, were often insufficiently detailed, thereby limiting comparability across the studies. Comparability was further hampered by the wide range of primary pathologies and applied RT modalities.

Similar to tumor-related epilepsy, the mechanisms underlying why some patients develop CRN-induced epilepsy, while others do not, remain poorly understood. The development of sCRN has been associated with a wide range of risk factors related to patient, tumor, and treatment characteristics. The single-center, retrospective study by Huang et al. (2020) [10] has built a model to predict CRN-induced epilepsy in NPC patients. The authors demonstrate that higher maximum radiation dose to the temporal lobe, larger RN volume, increased serum CK, lower cholesterol, a history of hypertension and/or diabetes, and male sex were all predictors for a higher risk of epilepsy [10]. Among these, serum CK, RT-related variables, and cholesterol levels have been examined most extensively. Serum CK has been associated with generalized tonic–clonic seizures due to muscle damage in both animal and clinical studies, yet its relation with sCRN remains unclear [38,39]. Huang et al. (2020) [10] hypothesize that blood–brain barrier (BBB) disruption caused by CRN may cause a release of CK as part of metabolic disturbances, thereby marking a greater risk of developing seizures. Next, considering the RT-related variables, the study of Rong et al. (2017) [11] reinforces the finding that irradiated tumor lesion volume and radiation dosage to the temporal lobe are potential risk factors for CRN-induced epilepsy [11]. The vulnerability of the temporal lobe to epileptogenesis may be triggered by higher maximum radiation doses, which can induce inflammatory responses in this region [10]. In addition, a larger irradiated lesion volume may reflect greater disruption of the BBB and increased neuroinflammation, both of which may contribute to seizure development [10]. Finally, in contrast to the findings of Huang et al. (2020) [10] who reported that lower cholesterol levels were associated with an increased risk of epilepsy, Rong et al. (2017) [11] observed that statin-induced cholesterol reduction was associated with a decreased risk of CRN-induced epilepsy [10,11]. Overall, few studies have investigated the impact of different RT modalities on seizure occurrence, emphasizing the need for further research into this topic.

On a molecular level, varying mechanisms can drive epileptogenesis, including ion and water imbalances, the formation of excitatory synapses, and the presence of proinflammatory cytokines [40]. Genetic factors also contribute to the pathogenesis of epilepsy [41]. For example, the deletion of the gene encoding for the actively regulated cytoskeletal (ARC) protein has been associated with epilepsy in rodents. Notably, irradiation has also been shown to reduce ARC protein expression in animal models, suggesting a potential link between the molecular pathways underlying CRN and epileptogenesis [42]. Beyond the aforementioned patient, tumor and treatment characteristics studied within the included literature of this scoping review, no literature was found to investigate the underlying biological mechanism making CRN cause seizures. Consequently, more research is needed to elucidate the pathophysiological mechanism behind CRN-induced epilepsy.

The management of CRN-induced epilepsy is highly undervalued in the current literature. Most relevant studies focus on the overall treatment of sCRN only. To the best of our knowledge, there has been no research conducted on the efficacy of ASMs for CRN-induced epilepsy. Furthermore, the direct effects of dexamethasone, bevacizumab, or LITT on CRN-induced epilepsy remain largely unexplored. Even though it might be considered self-evident that treating sCRN leads to symptom relief including seizure reduction or even seizure freedom, there is conflicting evidence. For instance, Hou et al. (2024) [12] found that bevacizumab reduced the prevalence of CRN-induced epilepsy, whereas Huang et al. (2020) [10] reported that bevacizumab treatment increased the incidence of seizures. These conflicting findings may stem from the differences in study populations and CRN diagnostic criteria. Hou et al. (2024) [12] specifically administered bevacizumab to treat CRN-induced epilepsy, in contrast to Huang et al. (2020) [10], who excluded patients from the analysis if they had seizures as the first symptom of CRN. Epilepsy observed after bevacizumab treatment in the latter study could therefore have been de novo instead of recurring, which may have influenced the observed prevalence trends. Bevacizumab is generally reserved for patients with a more severe form of CRN. Patients who have had prior treatment with bevacizumab may therefore have an increased incidence of epilepsy in subsequent follow-up. Nevertheless, Huang et al. (2020) [10] hypothesized that bevacizumab-induced reversible posterior leukoencephalopathy syndrome (RPLS) may be the underlying mechanism behind the increased occurrence of epilepsy, although they did not further investigate this theory [10,12]. Finally, Hou et al. (2024) [12] did not specify the criteria they used to diagnose CRN, which may have further limited comparability with Huang et al. A recent article by Jung et al. (2025) [43], published after our initial search, examined CRN-induced epilepsy as a secondary focus and found that bevacizumab did not worsen seizures and generally improved sCRN. On the whole, bevacizumab is recognized to improve CRN-induced symptoms [43,44,45]. However, whether bevacizumab is beneficial for CRN-induced epilepsy, remains to be established.

Overall, management of CRN-induced epilepsy should always involve both seizure management with ASMs, and the treatment of the underlying CRN. Currently, sCRN is primarily managed with corticosteroids, while bevacizumab is used when corticosteroids cause severe side effects, provide insufficient symptom control, or interfere with immunotherapy. Surgery is reserved for select cases, such as those with significant mass effect due to CRN. Other therapeutic options, such as LITT and hyperbaric oxygen treatment (HBOT), are less frequently used in routine practice but are increasingly studied [45]. The optimal treatment strategies for sCRN and CRN-induced epilepsy need to be further addressed in future studies. The primary studies on CRN-induced epilepsy included in this review reported follow-up periods ranging from approximately 1 to 3 years. Evidence on longer-term seizure outcomes is currently lacking. The listed studies provide some insight into long-term treatment outcomes, including the effects of statins and bevacizumab, but their focus varies and the available evidence for the longer term remains limited.

A key strength of this review is that it is the first to specifically address CRN-induced epilepsy. This review provides valuable insights into this condition, which is of growing relevance given the rising incidence of delayed RT toxicities. Nevertheless, several limitations should be acknowledged. First, the literature selection and data extraction were initially performed by a single author due to time and resource constraints. However, a second reviewer verified all excluded studies, and expert consultation was sought to further identify any remaining relevant literature. As we did not implement an initial independent dual screening approach, a certain risk of selection bias may still be present, although reproducibility was partially safeguarded by the verification round of the second reviewer. Second, only two databases were used to identify relevant articles, again due to time and resource constraints, which may potentially have limited the completeness of our search. Nevertheless, this limitation was partly mitigated by additional screening of reference lists, although it may not have been fully eliminated.

Altogether, this scoping review provides a comprehensive overview of the current literature on CRN-induced epilepsy, capturing the main developments in the field and giving new perspectives for future research. Within the management of sCRN, the improvement of clinical outcomes and mitigation of severe clinical symptoms, such as epilepsy, should be prioritized. Future clinical trials in patients with sCRN, such as the BRAINS trial (ClinicalTrials.gov: NCT06888817), may shed more light on the clinical effectiveness of systemic treatment with bevacizumab, including on specific clinical outcomes such as daily functioning, quality of life, and seizure control [45].

## 5. Conclusions

This scoping review shows that the available literature on CRN-induced epilepsy is both scarce and heterogeneous. Only 3 of the 24 included studies focused on CRN-induced epilepsy as a primary outcome. Moreover, the included studies showed significant variation in primary pathology, diagnostic criteria for CRN, and RT modalities applied. These inconsistencies complicate research reproducibility and comparability across studies. Overall, CRN-induced epilepsy remains a largely underexplored topic, warranting the need for further robust clinical research aimed at providing evidence that can directly benefit patients affected by sCRN.

## Figures and Tables

**Table 1:** Explanation of study characteristics.

Data Item	Description	Outcome
Study aim	Central aim of the article	Outline of study aim
Article focus	CRN epilepsy as primary, secondary or exploratory focus	Primary, secondary or exploratory
CRN patients	Number of patients with CRN within the study	Number and percentage
CRN-epilepsy	Number of patients with CRN-induced epilepsy	Number and percentage
CRN diagnosis	CRN diagnostic modality	Diagnostic modality
CRN time interval	Time interval between last RT and CRN diagnosis	Time in months
RT modality	RT-modality and radiation dosage	RT modality and dosage
Seizure type	Seizure types, i.e., focal or generalized seizures	Distribution of seizure types
Seizure focus	Location of CRN (probably) resulting in epilepsy	Distribution of CRN locations

Abbreviations: Cerebral radiation necrosis (CRN) and radiotherapy (RT).

**Table 2:** General characteristics of the included studies.

	Author	Year	Country	Design	Approach	Cohort Size (F%)	Primary Diagnosis
PubMed & Embase	Alfotih [17]	2016	China	Observational	Longitudinal	12 (0%)	NPC
	Bhansali [18]	2004	India	Observational	Longitudinal	11 (18%)	Pituitary adenoma
	Black [19]	2006	USA	Observational	Longitudinal	11 (45%)	Mixed pathologies
	Black [20]	2013	USA	Observational	Longitudinal	2 (100%)	Astrocytoma
	Blakstad [21]	2022	Norway	Observational	Longitudinal	2 (50%)	Mixed pathologies
	Calafiore [22]	2025	USA	Observational	Longitudinal	14 (29%)	Meningioma
	Chan [23]	2023	USA	Observational	Longitudinal	90 (58%)	BMs
	Di Stefano [24]	2019	Italy	Observational	Longitudinal	26 (19%)	Mixed pathologies
	Fan [25]	2018	USA	Observational	Longitudinal	6 (33%)	Mixed pathologies
	Ferlazzo [26]	2018	Italy	Observational	Longitudinal	2 (0%)	Medulloblastoma
	Goertz [27]	2024	Germany	Observational	Longitudinal	44 (82%)	Meningioma
	Hilard [28]	2003	USA	Observational	Longitudinal	10 (90%)	Mixed pathologies
	Holt [29]	2015	USA	Observational	Longitudinal	13 (62%)	BMs
	Hou [12]	2024	China	Observational	Longitudinal	41 (51%)	Meningioma
	Huang [10]	2020	China	Observational	Longitudinal	302 (21%)	NPC
	Kawamura [30]	2012	Japan	Observational	Longitudinal	11 (91%)	MTLE
	Lau [31]	2014	USA	Observational	Longitudinal	100 (58%)	BMs
	Rong [11]	2017	China	Observational	Longitudinal	532 (24%)	NPC
	Shah [32]	2025	USA	Observational	Longitudinal	55 (49%)	BMs
	Vaios [33]	2022	USA	Observational	Longitudinal	2 (50%)	Lymphoma
	Wang [34]	2012	Taiwan	Observational	Longitudinal	37 (49%)	BMs
	Woo [35]	1987	China	Observational	Longitudinal	7 (29%)	NPC
Other	Minniti [36]	2014	Italy	Observational	Longitudinal	135 (47%)	BMs
	Sujijantarat [37]	2020	USA	Observational	Longitudinal	38 (50%)	BMs

Abbreviations: Female (F), asopharyngeal carcinoma (NPC), brain metastases (BMs), and mesial temporal lobe epilepsy (MTLE).

**Table 3:** Scope of literature with regard to CRN-induced epilepsy.

Author		Study Aim	CRN Patients (% of Total Cohort Size)	CRN-Induced Epilepsy (% of CRN Patients)
Primary	Hou [12]	Evaluating the use of bevacizumab for epilepsy after RT in meningioma patients	41 (100%)	41 (100%)
	Huang [10]	Developing a nomogram to predict epilepsy in NPC patients with CRN	302 (100%)	65 (22%)
	Rong [11]	Clarifying the use of statins on preventing CRN-induced epilepsy in NPC patients	471 (89%)	36 (8%)
Exploratory	Alfotih [17]	Presenting experience with surgery on temporal lobe CRN in NPC patients	12 (100%)	2 (17%)
	Bhansali [18]	Evaluating the profile of pituitary macroadenoma treated with RT	11 (100%)	4 (36%)
	Black [20]	Reviewing clinical findings in patients with SMART	11 (100%)	9 (82%)
	Calafiore [22]	Improving local control of recurrent meningiomas with a RT boost	1 (7%)	1 (100%)
	Chan [23]	Exploring the use of LITT for the treatment of CRN in BM patients	90 (100%)	31 (34%)
	Di Stefano [24]	Investigating the outcomes and treatment of stroke-like events after RT to the brain	26 (100%)	9 (35%)
	Goertz [27]	Evaluating postoperative seizures in patients with posterior fossa meningioma	1 (2.3%)	1 (100%)
	Hilard [28]	Reviewing the effects of multiple RT treatments and related toxicities	1 (10%)	1 (100%)
	Holt [29]	Evaluating outcomes of patients with BM treated with SRT, surgery and repeat SRT	2 (15%)	1 (50%)
	Kawamura [30]	Reporting on long-term seizure outcome and AEs of RT for MTLE	1 (9%)	1 (100%)
	Lau [31]	Describing the clinical experience with SRT for multiple BMs	2 (2%)	1 (50%)
	Minniti [36]	Assessing three-fraction SRT for BMs and its prognostic factors for treatment response	12 (9%)	4 (33%)
	Shah [32]	Comparing CPB irradiation vs. SRT for resected BMs	CPB: 0 (0%) SRT: 7 (22%)	CPB: 0 (0%) SRT: 1 (14%)
	Sujijantarat [37]	Comparing LITT and bevacizumab for the treatment of CRN in BM patients	38 (100%)	5 (13%)
	Wang [34]	Presenting experience with hypofractionated SRT for resected BMs	1 (3%)	1 (100%)
Small Case Series	Black [19]	Describing two adults with SMART after RT	2 (100%)	1 (50%)
	Blakstad [21]	Presenting two adults with SMART syndrome	2 (100%)	2 (100%)
	Fan [25]	Investigating the association of SMART syndrome and seizures	6 (100%)	5 (83%)
	Ferlazzo [26]	Describing two SMART cases, reinforcing its seizure-dominant presentation	2 (100%)	2 (100%)
	Vaios [33]	Reporting on the resolution of CRN in two lymphoma patients after bevacizumab	2 (100%)	1 (50%)
	Woo [35]	Describing benign cases of CRN after RT for NPC	7 (100%)	6 (86%)

Abbreviations: Radiotherapy (RT), cerebral radiation necrosis (CRN), nasopharyngeal carcinoma (NPC), stroke-like migraine attacks after radiation therapy (SMART), laser interstitial thermal therapy (LITT), brain metastasis (BM), stereotactic radiotherapy (SRT), adverse events (AEs), mesial temporal lobe epilepsy (MTLE), and conformal partial brain (CPB).

**Table 4:** Clinical and methodological characteristics related to CRN-induced epilepsy.

Author	CRN Diagnosis	CRN Time Interval (Months)	RT Modality (Average Dose)	Seizure Type	Seizure Focus (% of CRN-Epilepsy)
Hou [12]	NR	NR	SRT (NR)	Focal only: 28 Generalized: 13	NR
Huang [10]	Clinical and radiological	12	Conventional RT or IMRT (NR)	NR	NR
Rong [11]	Clinical and radiological	24	Conventional RT or IMRT (NR)	NR	NR
Alfotih [17]	Histopathological	* 91	* Conventional RT or IMRT (66 Gy and 72 Gy)	NR	Temporal: 2 (100%)
Bhansali [18]	Clinical and radiological	46.8	Conventional RT (51.3 Gy)	Focal only: 0 Generalized: 4	NR
Black [20]	Clinical and radiological	240	NR	Focal only: 8 Generalized: 1	Parietal: 2 (44.4%) Frontal: 1 (11.1%) Temporal: 5 (11.1%) Unknown: 1 (11.1%)
Calafiore [22]	Clinical and radiological	NR	* Fractionated EBRT (25.2 Gy)	NR	NR
Chan [23]	Histopathological	NR	SRT and/or WBRT (NR)	NR	NR
Di Stefano [24]	Clinical and radiological	120	Conventional RT or WBRT (NR)	Focal only: 9 Generalized: 0	Parietal: 4 (44.4%) Frontal: 1 (11.1%) Temporal: 1 (11.1%) Cerebellum: 2 (22.3%) Unknown: 1 (11.1%)
Goertz [27]	Clinical and radiological	19	NR	Focal only: 1 Generalized: 0	Cerebral: 1 (2.3%)
Hilard [28]	Clinical and radiological	* 12	* WBRT and SRT (33.4 Gy)	Focal only: 1 Generalized: 0	Frontal: 1 (100%)
Holt [29]	Clinical and radiological	6	Fractionated SRT (42 Gy)	NR	Temporal: 1 (100%)
Kawamura [30]	Clinical and radiological	* 60	* SRT (20 Gy)	Focal only: 1 Generalized: 0	Temporal: 1 (100%)
Lau [31]	Histopathological	* 18	* SRT (18 Gy)	NR	NR
Minniti [36]	Clinical and radiological	8	Fractionated SRT (27–36 Gy)	NR	NR
Shah [32]	Clinical and radiological	NR	Fractionated SRT (24 Gy) Fractionated CPB (33 Gy)	NR	NR
Sujijantarat [37]	Varied per subgroup	3	SRT or SRT and WBRT (15–30 Gy)	NR	NR
Wang [34]	Histopathological	* 9	* WBRT and Fractionated SRT (24 Gy SRT only)	NR	NR
Black [19]	Clinical and radiological	* 180	* Fractionated SRT (64 Gy)	Focal only: 1 Generalized: 0	Temporal: 1 (100%)
Blakstad [21]	Clinical and radiological	* 132	* Fractionated SRT (54 Gy and 60 Gy)	Focal only: 2 Generalized: 0	NR
Fan [25]	Clinical and radiological	206	NR	Focal only: 2 Generalized: 3	Parietal: 3 (60%) Temporal: 2 (40%)
Ferlazzo [26]	Clinical and radiological	NR	Conventional RT (34 Gy and 54 Gy)	Focal only: 2 Generalized: 0	Occipital: 2 (100%)
Vaios [33]	Histopathological	* 11	* WBRT and SRT (12.5 Gy)	Focal only: 0 Generalized: 1	Frontal: 1 (100%)
Woo [35]	Clinical and radiological	60	Fractionated SRT (50.4–103.2 Gy)	Focal only: 5 Generalized: 1	Temporal: 6 (100%)

Abbreviations: Not reported (NR), Cerebral radiation necrosis (CRN), radiotherapy (RT), stereotactic radiotherapy (SRT), intensity-modulated radiotherapy (IMRT), external beam radiotherapy (EBRT), whole brain radiotherapy (WBRT), and conformal partial brain (CPB). Data obtained from individual patients with CRN-induced epilepsy rather than a study average (*).

## Data Availability

Data is contained within the article or Supplementary Material.

## Supplementary Materials

The following supporting information can be downloaded at: https://www.mdpi.com/article/10.3390/medicina62030561/s1, Supplementary S1: Preferred Reporting Items for Systematic reviews and Meta-Analyses extension for Scoping Reviews (PRISMA-ScR) Checklist; Supplementary S2: Search queries for PubMed and Embase; Supplementary S3: Microsoft Excel datasheet Studies that were excluded following full-text screening are cited in the supplementary materials (Supplementary S3) [7,13,14,46,47,48,49,50,51,52,53,54,55,56,57,58,59,60,61,62,63,64,65,66,67,68,69,70,71,72,73,74,75,76,77,78,79,80,81,82,83,84,85,86,87,88,89,90,91,92,93,94,95,96,97,98,99,100,101,102,103,104,105,106,107].

## Abbreviations

The following abbreviations are used in this manuscript:

AE	Adverse event
ALERT	Acute late-onset encephalopathy after radiation therapy
ASM	Antiseizure medication
BM	Brain metastasis
BBB	Blood–brain barrier
CK	Creatine phosphokinase
CPB	Conformal partial brain
CRN	Cerebral radiation necrosis
CRT	Conformal RT
CTCAE	Common Terminology Criteria for Adverse Events
EBRT	External beam radiotherapy
HBOT	Hyperbaric oxygen treatment
IMRT	Intensity-modulated radiotherapy
LITT	Laser interstitial thermal therapy
MTLE	Mesial temporal lobe epilepsy
NPC	Nasopharyngeal carcinoma
PIPG	Peri-ictal pseudoprogression
PNET	Primitive neuroectodermal tumor
RT	Radiotherapy
RPLS	Reversible posterior leukoencephalopathy syndrome
sCRN	Symptomatic cerebral radiation necrosis
SMART	Stroke-like migraine attacks after radiation therapy
SRT	Stereotactic radiotherapy
VMAT	Volumetric modulated arc therapy
WBRT	Whole-brain radiotherapy
